# Effects of chronic carbon monoxide exposure on fetal growth and development in mice

**DOI:** 10.1186/1471-2393-11-101

**Published:** 2011-12-14

**Authors:** Carolina C Venditti, Richard Casselman, Graeme N Smith

**Affiliations:** 1 Biomedical and Molecular Sciences, Queen's University, Kingston, ON, Canada; 2 Obstetrics and Gynecology, Kingston General Hospital, Kingston, ON, Canada

## Abstract

**Background:**

Carbon monoxide (CO) is produced endogenously, and can also be acquired from many exogenous sources: ie. cigarette smoking, automobile exhaust. Although toxic at high levels, low level production or exposure lends to normal physiologic functions: smooth muscle cell relaxation, control of vascular tone, platelet aggregation, anti- inflammatory and anti-apoptotic events. In pregnancy, it is unclear at what level maternal CO exposure becomes toxic to the fetus. In this study, we hypothesized that CO would be embryotoxic, and we sought to determine at what level of chronic CO exposure in pregnancy embryo/fetotoxic effects are observed.

**Methods:**

Pregnant CD1 mice were exposed to continuous levels of CO (0 to 400 ppm) from conception to gestation day 17. The effect on fetal/placental growth and development, and fetal/maternal CO concentrations were determined.

**Results:**

Maternal and fetal CO blood concentrations ranged from 1.12- 15.6 percent carboxyhemoglobin (%COHb) and 1.0- 28.6%COHb, respectively. No significant difference was observed in placental histological morphology or in placental mass with any CO exposure. At 400 ppm CO vs. control, decreased litter size and fetal mass (p < 0.05), increased fetal early/late gestational deaths (p < 0.05), and increased CO content in the placenta and the maternal spleen, heart, liver, kidney and lung (p < 0.05) were observed.

**Conclusions:**

Exposure to levels at or below 300 ppm CO throughout pregnancy has little demonstrable effect on fetal growth and development in the mouse.

## Background

At very high levels, carbon monoxide (CO) is a toxic gas; exogenous inhalation of CO levels higher than 3% (30 000 ppm) leads to lethal outcomes [1], but this is usually through accidental inhalation of smoke or exhaust in enclosed spaces [2]. Interestingly, CO is produced endogenously at low concentrations with normal physiologic functions: smooth muscle cell relaxation, control of vascular tone [3], platelet aggregation [4], anti-inflammatory and anti-apoptotic events [5].

Endogenously, the oxidization of heme by the enzyme heme oxygenase (HO) produces equimolar amounts of CO, iron and biliverdin [6]. Heme is a major structural component of hemoglobin (Hb), and the abundance of this molecule in red blood cells allows for the largest amount of heme for degradation. Additionally, CO has been measured in a number of tissues; most abundantly in muscle, heart, liver, spleen, and kidney [7]; although both endogenous levels and functionality are not well understood.

Exogenous inhalation of CO leads to its preferential binding with Hb, producing carboxyhemoglobin (COHb). This binding is in direct competition with molecular oxygen (O_2_), and binds to Hb with 250 times greater affinity than O_2 _[8]. This effect shifts the O_2 _dissociation curve to the left [9], limiting the release of O_2 _to the tissues, and can lead to hypoxia, or further, the asphyxiating effects of CO poisoning. Measurable CO levels in non- smoking adults are 0 to 1.5% COHb [10], whereas people who smoke may increase their %COHb levels to 14% [11,12]. Refer to Table 1 for a comparison of reference CO levels.

**Table 1:** Reference Carbon Monoxide levels in humans and various environmental sources

Subject/Source	CO level	Source
**Non smoker**	0-1.5% COHb	[10]

**Smoker**	Up to 14%COHb	[11,12]

		

**Natural urban air level**	1-30 ppm	[15]

**Levels found in homes**	0.5- 5 ppm	[42]

**Cigarette Smoke**	20 000-60 000 ppm	[15]

**Alveolar Concentration in smoker**	300-400 ppm	[15]

**Car Exhaust without catalytic converter**	30 000- 60 000 ppm	[15]

*Parts per million (ppm) by volume (100 ppm = 0.01% of CO in the air)

*Percent carboxyhemoglobin (%COHb) is the amount of CO bound to total haemoglobin

Although many negative effects are associated with cigarette smoking in pregnancy, 17% of Canadian women smoked cigarettes in their pregnancy between 1995 and 2001 [13]. Of the greater than 4800 toxic chemicals identified within cigarette smoke [14], a major combustible product is CO. It readily crosses the placenta and can combine with fetal Hb and tissue heme moieties [15]. When pregnant sheep were exposed to CO, a lag period was found before fetal CO levels began to rise, but shortly afterwards, they matched and even surpassed those of their mother. Fetal hypoxia may result at high levels of maternal CO exposure, however, the level at which maternal CO exposure becomes a fetal threat is unknown.

*In vitro, *researchers have isolated HO in human placental and umbilical cord tissues [16], suggesting that CO is produced in this tissue. We have shown that CO, at levels similar to those found in umbilical cord blood at delivery, is capable of placental vasorelaxation [17]. As the levels of CO in pregnant women who smoke are increased, this suggests an exacerbation of placental vasorelaxation, while we have shown that nicotine has no effect on the basal feto/placental perfusion pressure [18]. These results suggest that CO may play a role in placental development and/or its regulation of placental hemodynamics [19].

A few studies have examined the effects of CO exposure in pregnancy, but they yield conflicting results in different animal species [20-23]. Studies conducted in pregnant wister rats exposed dams to one CO dose above 1000 ppm for varying lengths of time throughout pregnancy, observing decreased fetal weights and litter size [20,21]. It is likely at such high exposures that CO poisoning occurred, however venous CO levels (57% COHb recorded) were measured only in one of the studies, with no mention of the method by which this number was obtained [21]. Studies conducted at 500 ppm CO and below [22-24] yield maternal %COHb levels of roughly 28 [7], approximating more closely %COHb of adults smoking roughly two packs of cigarettes/day (up to 14%) [25]. The results from each of these studies are difficult to compare, as dosing mechanisms, lengths and amounts of CO concentrations given to the animals vary between groups.

In the present study, we hypothesized that CO would be embryotoxic. We investigated the effect of chronic CO exposure to mice in pregnancy, and sought the level at which no maternal or feto/embryotoxic effects were demonstrable.

## Methods

### Ethics Statement

Experimental procedures were carried out according to the University Animal Care Committee of Queen's University and was approved by the Queen's University Ethics Committee (REB no. Smith 2007-052- Or).

### Animals and Husbandry

Timed matings of CD1 mice (Charles River, USA), female (6-8 weeks old) and males (5-7 weeks old), were performed overnight. Females were weighed and placed into a regulated CO-dosing chamber on GD1 (morning detection of a vaginal plug). Food and water were provided *ad libitum*.

### Carbon Monoxide Concentrations

Concentrations of CO administered were 0, 25, 60, 100, 150, 200 250, 300 and 400 ppm in ambient air. At each concentration a minimum of six pregnant female mice were utilized. Room air was collected using a compressor (Panther Compact 106, Silent Air Compressor, Texas, USA) and passed through a Norgren air dryer (Littleton, CO USA). The dry air was mixed with 10%CO gas (Praxair, Kingston, ON) using flow meters (Alicat Scientific, Tucson, AZ USA), and adjusted to the specific CO concentration required, using a Gas Mixing software Program (Qubit Systems Inc, Kingston, ON). The air was bubbled through distilled water, reintroducing humidity levels of 40-50% (animal care regulations), and monitored using a Humidity Sensor (Vernier relative humidity sensor, Mississauga, ON, CAN) and its software (Vernier Logger Lite Software, Mississauga, ON, CAN). This sensor also ensured the cage temperature was maintained at 25°C. The air mixture was vented into a tightly- sealed plastic aquarium, in which the mouse cage was placed, and exhausted out of the chamber into the room's air filter system. The chamber air was changed a minimum of 10 times per hour (flow rate 7000 ml/min). The cage was changed twice weekly by research personnel, at which time the CO administration was stopped (20 minute maximum). Air samples were collected at least twice weekly from the aquarium air- port, and measured using a gas-solid chromatography machine (GC) (Peak Performer 1, Palo Alto, San Francisco, USA), as previously described[26], ensuring CO levels matched the desired concentrations.

### Experimental Procedure

Female mice were anesthetised on GD17 by intraperitoneal injection of 10 mg/g of 2-2-2-Tribromoethanol (T48402, Sigma Aldrich, Canada). Upon reaching a surgical plane of anaesthesia, maternal blood was drawn by retro-orbital blood collection using a glass pipette, transferred to a microcentrifuge tube containing 10 microliter (μl) of 1430 U/ml sodium heparin (Sigma Aldrich, H0777), and placed immediately on ice. Mice were then perfused (gravity perfusion) through the left ventricle for 15 minutes with either phosphate buffered saline (PBS) (minimum of four mice/experiment) or 4% paraformaldehyde (PFA) (minimum two mice/experiment).

### Blood CO measurement

Hemoglobin was measured within 5 minutes of blood collection using a Hemocue Hb 201 (Hemocue, Sweden). Amber vials (2 ml) (Sigma- Aldrich Ltd) capped (Chromatographic Specialties C223710C) with 8 mm silica septa (Chromatographic Specialties, C13302) and containing 20 μl of 2% sulfosalycylic acid (Sigma Aldrich, Cat no. 86193) were purged with 21%O_2_/5%CO_2_/balance N_2 _(Praxair, Kingston, ON) sent through a catalytic converter (to reduce any CO present). Between 0.1 μl and 1 μl of blood (depending on the level of CO administration) was added using a gas tight Hamilton syringe and repeater system (Hamilton, USA). Triplicate vials per blood sample were prepared and set on ice for 60-120 minutes. Carbon monoxide levels were read using a GC and expressed as a percentage of total Hb using the following equation:

(1) %COHb=(volCO∕(Hb*1.368)]*100%[10]

where "vol CO" is milliliters of CO bound to 1L of blood, Hb is total Hb concentration in the blood (g/L) and 1.368 is the CO-binding capacity of Hb in millilitres per gram.

### PBS perfused mice

**Figure 1:**
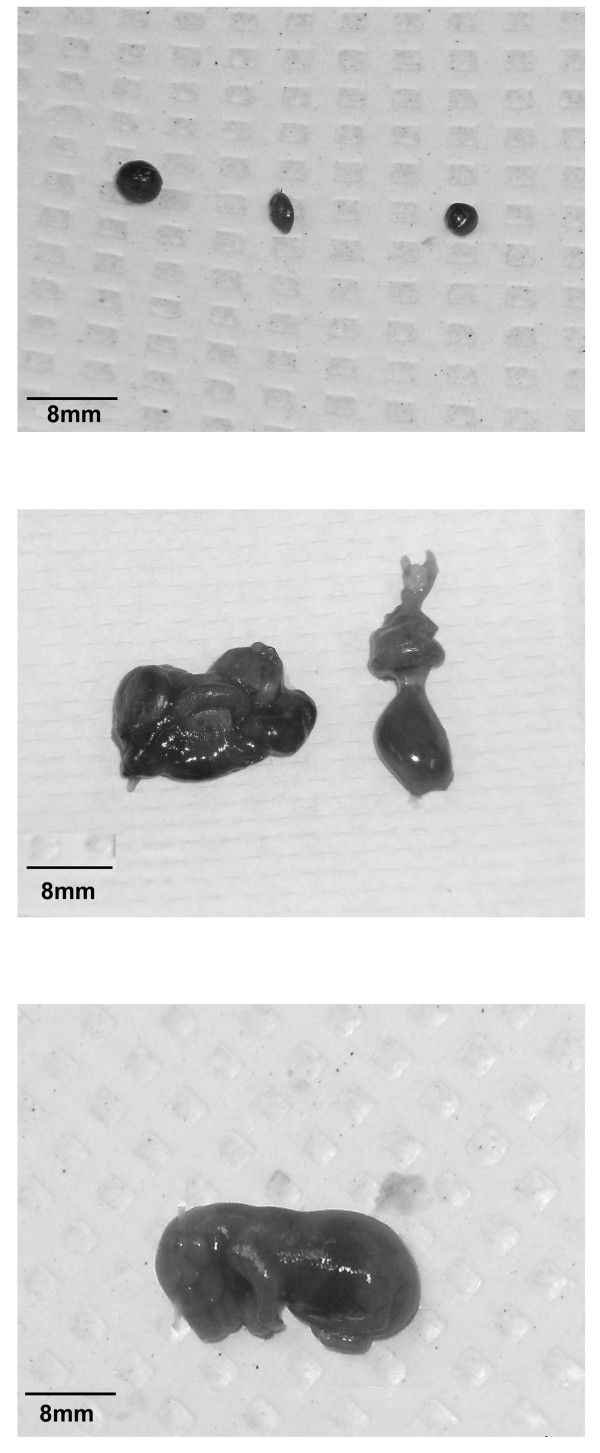
**Representation of fetal early/late gestational death**. A typical fetal early gestational demise is shown in A, a fetal late gestational death is shown in B, a normal fetus at GD17 is shown in C.

Following a 15 minute perfusion, the uterus was dissected out, and all fetuses and their respective placentas (excluding gestational losses) were removed and weighed. Litter size was noted for each mouse. All fetuses and placentas were examined for gross morphological abnormalities. Fetal mortality was classified as early gestational demise (EGD), or as a late gestational death (LGD) (Figure 1). We classified EGD as a resorbed fetus, where no separate distinction existed between placenta and fetus (Figure 1A), while LGD was determined by a discernible fetus and placenta (Figure 1B). A normal fetus at gestation day 17 is shown in Figure 1C.

### Fetal Blood Collection

Three random fetuses were decapitated and blood was collected using a heparin-coated capillary tube (Fisher brand, Cat no. 22-260-950). The blood from the fetuses was pooled into a microcentrifuge tube and blood processing was completed as previously described for the dams.

### Tissue CO Levels

Sections of maternal organs (brain, liver, lung, kidney, spleen, heart) and placenta were collected and placed on ice immediately. Tissue CO measurement was performed as per Vreman *et al*.[7].

### 4% PFA perfused mice

A minimum of 2 mice per experiment were perfused with PFA. Following perfusion with 4% PFA, the uterus was removed. Dissecting between implantation sites, three embryos were removed from each mouse's uterus, stored in 4% PFA for 24 hours, and transferred to 70% ethanol until processing. Embryos were embedded in paraffin according to standard procedures.

Each embryo (six per CO concentration) was sectioned (0.4 μm thickness) a minimum of 10 times in a saggital plane and placed on slides. Of the 10 slides, a random selection of 5 slides were chosen and stained with hematoxylin and eosin. Placentas were analyzed for histological morphometric changes compared to control. Three pictures were taken using Nikon Eclipse E800 Light Microscope and Q capture software of the whole placenta (10X and 40X magnification) as well as the placenta labyrinth (200X magnification). The placentas were analyzed for relative proportions of labyrinth versus junctional zone and alterations in cell shape and size. A third party anatomist was consulted on all histological slides analyses.

### Statistical Analysis

Dose response curves for maternal/fetal %COHb and Hb concentration were analyzed using linear regression analysis. The EGD and LGD data was analyzed with a chi squared test using the fisher exact method. EGD were compared to total implantation sites subtract EGD and LGD, while LGD were compared to live implantation sites subtract LGD. For this test, OpenEpi.com version 2.3.1 was used to analyze data. All other analyses were completed using a one way analysis of variance with a post-hoc Dunnett's multiple comparison test, using Graphpad prism version 6. A p-value less than 0.05 was deemed significant for all tests. All statistical results are reported with standard deviation.

## Results

All of the required CO levels for this study were achieved using the computerized software and were confirmed using GC to be between 0.5 and 5.0 ppm (up to 1.6%) of the desired CO levels.

For each CO exposure, a minimum of four mice were perfused with PBS and two mice with PFA. The amount of litters per experimental group for the exposures of 0, 25 and 60 ppm CO are larger than the remaining exposures. This was due to a slight change in protocol following the beginning of the study, where we developed a method to measure fetal blood CO levels. Therefore, we repeated the first three experiments at 0, 25 and 60 ppm CO, in order to acquire this data.

**Figure 2:**
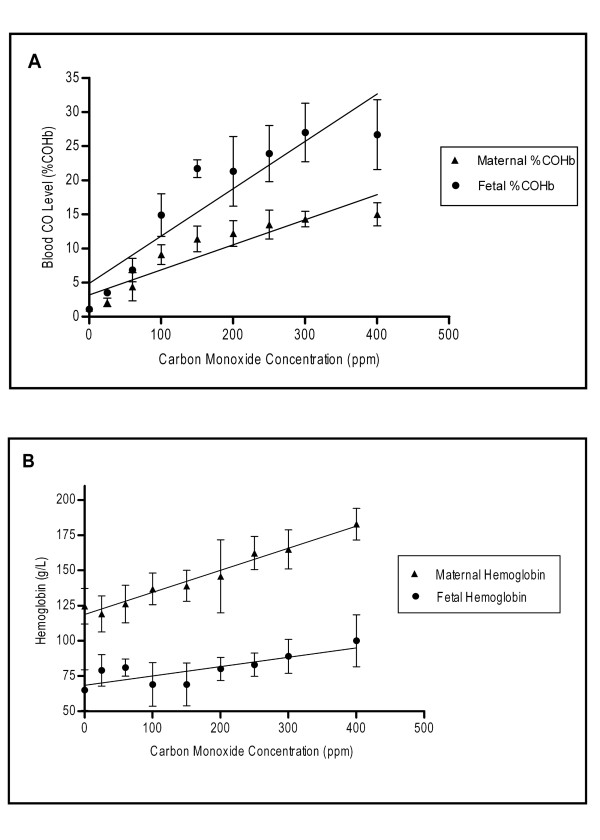
**Dose responses of maternal/fetal Hb and CO levels to increasing maternal exogenous CO exposure**. A positive trend was observed with both maternal and fetal blood %COHb vs. CO concentration exposure (A). A positive trend was observed with both maternal and fetal Hb vs. CO concentration exposure (B).The slope of the line significantly deviated from zero in both cases, A and B (p < 0.05).

The blood %COHb levels were positively correlated with increasing CO concentration exposure, for both maternal (p < 0.01) and fetal (p < 0.01) measurements (Figure 2A). The ratio of fetal/maternal %COHb was a mean (SD) of 1.74X (0.12), beyond 25 ppm. Additionally, Hb followed the same trend as %COHb levels, and was positively correlated with increasing CO concentrations for both maternal (p < 0.01) and fetal (p < 0.01) levels (Figure 2B).

**Figure 3:**
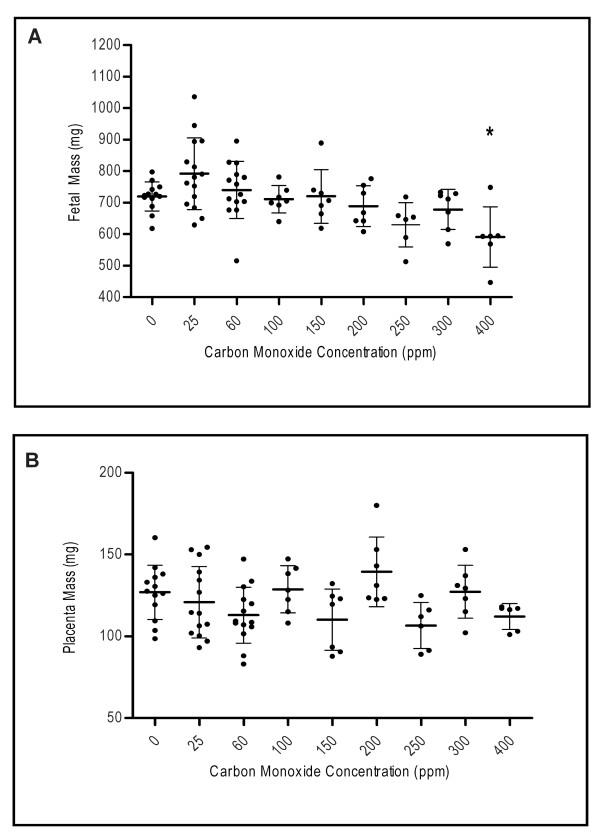
**Comparison of mean fetal and placental mass to maternal exogenous CO exposure**. Mean fetal mass for each CO exposure level is displayed in figure A, with a significant decrease in mass observed only at 400 ppm (* p < 0.05). Mean placental mass for each CO exposure level is displayed in figure B; no significance at any of the CO exposures was observed (p > 0.05).

Mean fetal mass did not differ compared to control with CO concentrations of 25 ppm to 300 ppm (Figure 3A). At 400 ppm CO, fetal mass was significantly lower than that of the control (p < 0.05). Placental mass was not found to be significantly different than the control with any of the CO doses (p > 0.05) (Figure 3B). Litter size was significantly reduced only at 400 ppm compared to control (p < 0.05) (Table 2).

**Table 2:** The effect of maternal CO exposure on total number of early gestational demise/late gestational death

CO (ppm)	Total Implantation sites/litter	Total live fetuses/litter	**EGD (%)	RR of EGD (CI)	**LGD (%)	RR of LGD (CI)
**0**	12.8	12.6	1.12		0.56	

**25**	12.2	11.9	2.40	2.15 (0.42, 10.95)	0.48	0.86 (0.05, 13.66)

**60**	12.8	12.4	1.81	1.62 (0.27, 0.96)	1.20	2.16 (0.20, 23.56)

**100**	14.2	13.9	0.78	0.70 (0.06, 7.63)	1.56	1.40 (0.09, 22.15)

**150**	13.6	13.5	0.92	0.82 (0.08, 8.95)	0.00	0.00

**200**	10.9	10.5	1.15	1.03 (0.09, 11.19)	2.30	1.74 (0.38, 44.76)

**250**	12.9	12.4	2.91	2.67 (0.44, 15.34)	0.90	1.74 (0.11, 27.49)

**330**	13.9	12.1	8.80*	7.88 (1.78, 34.92)	4.00	7.16 (0.85, 60.54)

**400**	13.7	8.2*	13.40*	12.01 (2.72, 52.94)	26.83*	48.02 (6.59, 350.20)

EGD: early gestational demise LGD: late gestational death RR: relative risk

CI: confidence interval

Statistical Analysis: Chi squared test, using the fisher exact method to compare EGD or LGD to total implantation sites - EGD or LGD and analysis of variance for live fetuses/litter (*p < 0.05).

**Equations: EGD (%): Total EGD/(Total Implantation Sites- EGD)*100, LGD (%): Total LGD/[(Total Implantation Sites-(EGD + LGD)]*100

In Table 2, total implantation sites/litter did not differ between experimental CO exposures. The number of EGD and LGD increased with maternal CO exposure. EGD is presented as EGD/(total implantation sites- EGD) *100% and LGD is presented as LGD/[total implantation sites- (LGD + EGD)]*100%. A significant increase in number of fetal EGD was observed at 300 and 400 ppm, while LGD were found to be significant compared to control at 400 ppm (p < 0.05). Relative risk data and confidence intervals are listed in Table 2.

**Figure 4:**
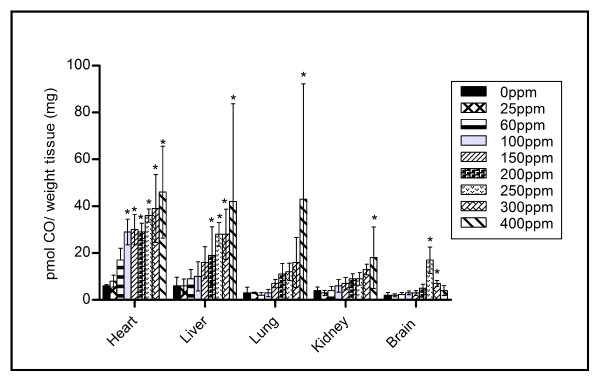
**Maternal tissue CO levels in heart, liver, lungs, kidney and brain for each CO exposure**. Significance was found in the heart tissue at CO levels ≥100 ppm, in the liver at CO levels ≥ 200 ppm, in both the lung and kidney at the CO level of 400 ppm only and finally in the brain at CO levels of 250 and 300 ppm only (* p < 0.05).

**Figure 5:**
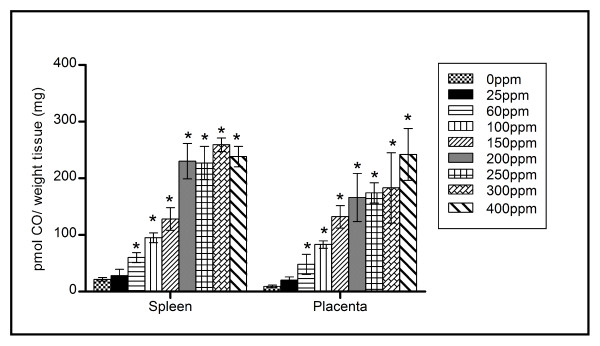
**Placenta and maternal spleen tissue CO levels with increasing CO exposure**. Compared to control, significance was observed in both placenta and spleen at CO levels ≥ 60 ppm exposure (* p < 0.05).

Figure 4 displays the tissue CO concentration in the maternal heart, liver, lung, kidney and brain, for each CO exposure. An elevation in tissue CO concentration is observed for most tissues, as maternal CO exposure increases. Figure 5 displays the CO levels in the placenta and maternal spleen for each CO exposure. These organs are shown separately as they contained at least four times the CO level as the other tissues examined. At 60 ppm CO exposure, significant differences (p < 0.05) are observed in both tissue samples, compared to control tissue.

Blinded histological analysis of placental tissue did not show any gross morphological differences between those exposed to CO and controls (data not shown). All parties analyzing histological slides noted no differences in relative amounts of labyrinth versus junctional zone. Additionally, no alterations in cell shape and size were observed.

## Discussion

A number of studies have evaluated the effects of CO exposure on pregnancy; however a direct comparison of these studies is difficult because of significant methodologic differences. For example, the method of CO delivery varied from whole body[20-22] to nose-only inhalation [27], time exposure (acute vs. chronic) differed; daily time intervals or specific days of gestation. The dosing concentrations differed, with some studies evaluating CO levels of 1000 ppm to 10 000 ppm [20,28], well above the toxic range. No previous study has correlated maternal/fetal CO levels with maternal/fetal outcomes. Our study's aim was to use a highly regulated CO chamber system to expose pregnant mice to chronic CO concentrations throughout gestation, that would mimic those of smoking adults (2-14%COHb)[25].

Maternal cigarette smoking is perhaps one of the most common chronic methods by which CO levels are increased, thereby exposing the fetus to greater than normal CO levels. Our laboratory has measured a range of COHb in maternal smokers of 1.5-9.85%COHb (unpublished), while a level of 14%COHb has been determined previously by other researchers [11,12]. Cigarette smoke contains an average of 4% or 40 000 ppm CO by volume [29], and during the smoking process it becomes diluted in air, leaving the alveoli to see roughly 400-500 ppm [15]. It is known that CO can cross the placenta, therefore CO can affect the fetal partial pressure of O_2 _(pO_2_) [15].

In the present study, the highest dose of CO yielded a mean maternal %COHb level of 15.6, while women who smoke heavily during pregnancy have a calculated CO level of up to 14%COHb [11,12]. It is important to note that in comparison with women who intermittently smoke, and thus are affected by peaks and troughs of high CO exposure, these mice were subjected to elevated CO concentrations on a constant basis. The half- life of CO is 2-3 hours when breathing room air [15], therefore a smoker would have peak CO levels directly following their smoking a cigarette, which would slowly dissipate until the next cigarette. Also of importance are the elevated ventilation and elimination rates, along with increased CO uptake, present in smaller mammals compared to humans [30]. As explained by Klimisch *et al.*[30], the higher ventilation rates by smaller mammals allow for a faster rate of Hb saturation by CO. Therefore, the %COHb results observed in this study are presumably higher than those that would be seen in women under similar exposure. The %COHb values cannot be evaluated directly, but can be used as representative values to compare the mouse model with the potential effects in pregnant women.

As an adaptation response to increased CO levels, likely in part due to hypoxia, it is documented that mammals will increase Hb concentration [31] in order to increase the O_2 _carrying capacity of the blood. A significant dose- response was observed with both the maternal and fetal Hb to increasing CO exposure levels, and one would expect a similar response in pregnant women.

The ratio between maternal and fetal %COHb concentrations (1.74) is in agreement with a study conducted by Longo and Hill [32], and similar to results recorded by Bureau *et al.*[33]who showed that the ratio of fetal/maternal COHb was 2.5 (using umbilical blood as a fetal representative value). The Hb levels of both maternal and fetal systems differ and the affinity of fetal Hb for CO is established to be higher than that of the maternal system [12]. This, coupled with the lower pO_2 _in capillary blood of the fetus versus maternal system [12], would add to the effect of increased fetal %COHb compared to maternal %COHb.

There is considerable variability in the effects of CO exposure on fetal birth weight in the published literature. It is well established that pregnant women who smoke cigarettes will give birth to a neonate roughly 200 g lighter (per pack per day smoked) than a fetus born to a non- smoking mother [34]. However, given that there are thousands of toxic substances in cigarette smoke, the specific role of CO in these situations cannot be determined. Wouters *et al *[28] and Soothill *et al.*[35] studied the human effects of increased fetal COHb on fetal weight, with conclusions in both cases that data was not convincing for a simple cause-effect relationship. Our study found a significant decrease in birth weight compared to control only at our highest CO dose. Other studies have also found a decrease in birth weight with increasing CO exposure [20-23]. Singh and Scott found that subjecting pregnant CD-1 mice (on GD 8 to GD 18) to CO exposures of 0 to 500 ppm (controlling chamber levels using a CO monitor), lead to a significant decrease in fetal weight as early as 125 ppm, but not in a dose dependent manner [24]. This study did not measure CO levels in either maternal or fetal mice. Given the toxic effects demonstrated by Singh and Scott as early as 125 ppm, which conflicts with our data, this presents a possibility of fetal adaptation to CO exposures when subjected from conception, rather than mid- way through pregnancy. Future studies would be necessary to further examine this hypothesis.

Interestingly, placental weight was not significantly different than the control at any of the CO doses. Bissonnette and Wickham [36] reported that placental CO diffusing capacity increased significantly with greater gestational age, and correlated with fetal weight but not placental weight. Thus, as CO levels increased across our experiment, no correlation was expected with placental weight. Litter size (number of healthy fetuses) however, was significantly decreased at 400 ppm CO exposure compared to control. In previous studies, litter size in rats was not shown to be affected when dams were chronically exposed to 150 ppm CO throughout gestation [37], but was decreased when dams were exposed to acute doses of 1000 to 1600 ppm twice daily throughout pregnancy [20]. An increase in EGD and LGD above 180 ppm maternal CO exposure has been reported previously in varying animal models [23,24], although CO exposure levels and dosing schedules were different amongst them. In our study, while EGD proved significant compared to control at 300 ppm, at 400 ppm, more fetuses appeared to have died in late gestation. As the placenta's diffusing capacity increases with gestational age [38], perhaps fetal health was more greatly compromised later in pregnancy with higher CO doses, as more potent CO was able to diffuse to the fetal circulation.

Maternal tissue CO levels proved that aside from Hb, the gas was also accumulating in the heme- containing molecules found throughout tissue. At 0 ppm CO, tissue CO levels closely approximated those previously reported [7]. Both the spleen and the placenta were the most difficult tissues to perfuse, as their open circulation proved difficult for the complete removal of blood. This would explain the much higher CO levels compared to the other tissues sampled. A dose response was observed in both tissues. Of the remaining tissues measured, the heart expressed the highest CO concentration, possibly due to high myoglobin content, a heme containing protein with an affinity for CO second only to Hb [39].

In this study, at 400 ppm maternal CO exposure, a number of toxic results were noted. Compared to control, both maternal and fetal %COHb and Hb values were significantly increased, fetal birth weight and litter size were significantly decreased, and EGD/LGD were significantly increased. Maternal tissue CO levels increased as CO doses were raised, lending to the highest measurable CO amounts at 400 ppm. These results indicate that the chronic CO exposure to pregnant mice, at levels above 300 ppm, results in a feto-toxic effect.

Recently, CO has come into light as a possible therapeutic, due to its many beneficial effects: endogenous and exogenous CO can promote angiogenesis[40], suppress the release of anti-angiogenic markers[41], decrease inflammation and apoptotic events[5], and vasodilate blood vessels[16]. These beneficial properties of CO may be helpful in the treatment of disorders concerning angiogenesis and vascularity, including those in pregnancy. This study is of great importance as we have identified a fetal toxic threshold of CO in pregnancy above 300 ppm maternal exposure. This information can be used in future experimental designs, when examining the potential beneficial properties of CO exposure to treat disorders of pregnancy.

## Conclusions

Our study indicates that exposure to levels at or below 300 ppm CO throughout pregnancy has little demonstrable effect on fetal growth and development in the mouse.

## Abbreviations

CO: carbon monoxide; %COHb: percent carboxyhemoglobin; HO: heme oxygenase; Hb: haemoglobin; O2: oxygen; GC: gas chromatography; μl: microliter; EGD: early gestational demise; LGD: late gestational death; PBS: phosphate buffered saline; PFA: paraformaldehyde.

## Competing interests

The authors declare that they have no competing interests.

## Authors' contributions

CCV, RC and GNS participated in the design of the study. CV and RC carried out all mouse procedures. CCV carried out all analysis and preparation of histology, blood and tissue samples. The manuscript was drafted by CCV and reviewed and edited by GNS. Statistical analysis was performed by CCV, and reviewed by GNS. All authors read and approved the manuscript.

## Pre-publication history

The pre-publication history for this paper can be accessed here:

http://www.biomedcentral.com/1471-2393/11/101/prepub
